# Sanyin decoction alleviates psoriasis by reshaping gut microbiota and modulating the gut–spleen–skin axis

**DOI:** 10.3389/fmicb.2026.1799928

**Published:** 2026-04-16

**Authors:** Huangchao Jia, Weiming Zhang, Zhibo Yang, Mengyue Xu, Xuewei Liu

**Affiliations:** 1 Department of Dermatology, The First Affiliated Hospital of Henan University of Chinese Medicine, Zhengzhou, Henan, China; 2 Department of Dermatology, Wuhan First Hospital, Wuhan, Hubei, China; 3 Department of Dermatology, Hunan Traditional Chinese Medical Hospital, Changsha, Hunan, China; 4 Henan University of Chinese Medicine, Zhengzhou, Henan, China

**Keywords:** gut microbiota, gut-spleen-skin-axis, IL-4, multiomics, psoriasis, sanyin decoction, short chain fatty acids

## Abstract

**Background:**

Sanyin decoction (SYD) is an improved formulation based on Xiaoyinsan, commonly used as an adjunct treatment for psoriasis; however, its molecular mechanisms remain unclear.

**Methods:**

A psoriasis-like mouse model was established using imiquimod induction. Mice were treated with SYD via gavage, and their health status was assessed based on skin histopathology, PASI scores, and ear thickness measurements. Fecal samples were collected for 16S rRNA sequencing and short-chain fatty acid (SCFA) analysis to examine changes in gut microbiota and SCFA levels before and after SYD treatment. Additionally, transcriptomic sequencing was performed on the spleen and skin lesions to analyze gene expression changes and immune modulation in these tissues.

**Results:**

SYD improved the skin pathology, reduced lesion scores, and decreased ear thickness in psoriasis-like mice. Following treatment, the gut microbiota of treated mice showed significant enrichment of short-chain fatty acid-producing bacteria, including *g__Lachnospiraceae, g__Rikenella*, and *g__Muribaculum*, with increased levels of SCFAs, particularly acetate, in the feces. Transcriptomic analysis of splenic tissue revealed a significant upregulation of IL-4 expression and activation of G protein–coupled receptor–related signaling pathways following treatment. In addition, the abundance of regulatory T cells in the spleen was significantly increased. Plasma IL-4 level was significantly higher after treatment. In lesional skin, SYD treatment was associated with significant suppression of the IL-17 signaling pathway, along with activation of keratinocyte differentiation-related pathways were activated, promoting tissue repair.

**Conclusion:**

Our study suggests that acetate derived from gut microbiota may serve as an important link between SYD treatment and systemic immune regulation in psoriasis, highlighting the potential of the gut–spleen–skin axis as a target for traditional herbal interventions.

## 1 Introduction

Psoriasis is a chronic, immune-mediated inflammatory skin disorder characterized by infiltration of various immune cells and excessive proliferation of keratinocytes (Griffiths et al., 2021; Nestle et al., 2009; Bos and De Rie, 1999). While skin manifestations are the most prominent feature, increasing evidence suggests that psoriasis is a systemic disease driven by complex interactions between innate and adaptive immunity (Bos and De Rie, 1999), with the IL-23/Th17 axis recognized as a central pathogenic feature (Varshney et al., 2016). In addition, psoriasis often coexists with metabolic syndrome, inflammatory bowel disease and cardiovascular disease, suggesting that its pathogenic process goes beyond the skin space and involves multiple organ systems (Parisi et al., 2020).

In recent years, gut microbiota has become an important regulator of immune and systemic inflammation (Di Vincenzo et al., 2024). Several clinical and experimental studies have reported significant alterations in the gut microbiota composition of psoriasis patients, including reduced microbial diversity and changes in specific bacterial groups associated with inflammatory responses (Buhas et al., 2022; Cai et al., 2023; Du et al., 2023; Giambra et al., 2023; Li et al., 2023). The current research emphasizes the key role of “gastrointestinal skin axis” in the pathogenesis of psoriasis (Mahmud et al., 2022). Compared to healthy individuals, psoriasis patients often exhibit pronounced gut dysbiosis, characterized by a decrease in beneficial symbionts and alterations in their metabolic profile (Zeng et al., 2021; Memariani and Memariani, 2025). Microbial-derived metabolites, particularly short-chain fatty acids (SCFAs) such as acetate, propionate, and butyrate, serve as key signaling molecules linking the gut to distal organs (Tan et al., 2014; Smith et al., 2013). Once in the bloodstream, SCFAs can exert effects both locally within the gut and systemically, influencing immune responses in distant organs (Thorburn et al., 2014). Specifically, acetate regulates systemic immune responses by modulating T cell differentiation and inhibiting the production of pro-inflammatory cytokines (Smith et al., 2013; Mandaliya et al., 2021; Al-Harbi et al., 2018; Qiu et al., 2019). While SCFAs have been extensively studied in the context of intestinal inflammation and metabolic diseases, their potential involvement in psoriasis, particularly through systemic immune modulation, has yet to be fully elucidated.

As the largest secondary lymphoid organ, the spleen is the main reservoir of immune cells and the key part to start the adaptive immune response (Lewis et al., 2019; Bronte and Pittet, 2013). It serves as a physiological “training ground,” where immune cells are primed before mi grating to peripheral tissues, including the skin. Emerging evidence suggests that gut-derived microbial metabolites can influence the composition and activation state of splenic immune cells, thereby forming downstream immune responses in peripheral tissues, including the skin (Provitera et al., 2024; Yoo et al., 2020; Stec et al., 2023). Therefore, the concept of the gut–spleen–skin axis provides a reasonable framework for explaining how changes in the gut microbiota may contribute to the systemic immune dysregulation and skin inflammation observed in psoriasis. However, direct experimental evidence supporting the involvement of this axis in the pathogenesis of psoriasis remains limited.

Traditional Chinese medicine has unique advantages in treating complex inflammatory diseases through multi-component and multi-target mechanisms. SYD is a clinical empirical formula with the effect of clearing heat and cooling blood, which is based on Xiaoyinsan, and Commonly used in the clinical treatment of psoriasis. Although it has been used in clinical practice, whether SYD can play its therapeutic role in reshaping intestinal flora and subsequently regulating the skin axis of intestine and spleen is still unknown.

In this study, we used an imiquimod (IMQ)-induced psoriasis-like mouse model to investigate the immunomodulatory mechanisms of SYD. By integrating 16S rDNA sequencing, targeted fecal SCFA metabolomics, and transcriptomic analysis of spleen and skin tissues, we aimed to verify the hypothesis that SYD alleviates psoriasis by reshaping the gut microbiota composition and enhancing acetate production. We further hypothesized that elevated acetate levels suppress excessive immune activation in the spleen, thereby reducing skin inflammation. This study provides new insights into the systemic regulatory mechanisms of SYD and highlights the potential of targeting the gut–spleen–skin axis for the treatment of psoriasis.

## 2 Materials and methods

### 2.1 Animal model construction and experimental design

Female BALB/c mice aged 6–8 weeks (body weight 18–22g) were obtained from a licensed laboratory animal supplier and housed under specific pathogen–free (SPF) conditions with a 12 h light/dark cycle and *ad libitum* access to food and water. All animal procedures were approved by the Institutional Animal Care and Use Committee of Wuhan Servicebio Technology Co., Ltd [approval No. Servicebio Dong (Fu) No.2025108] and were conducted in accordance with the ARRIVE guidelines. Animals were randomly assigned to three groups (*n* = 6 per group): control group, model group, and SYD treatment group. A psoriasis-like mouse model was established using 5% imiquimod cream (Aldara, 3M Pharmaceuticals) as the inducing agent, mice treated with vehicle cream containing a petrolatum base served as the healthy control group. After a 2-day acclimatization period in the SPF facility, the dorsal hair of the mice was shaved to prepare the application area. Imiquimod cream was topically applied once daily at a fixed time to the shaved dorsal skin at a dose of 62.5 mg/kg for 7 consecutive days, while control mice received an equivalent amount of vehicle cream. Successful establishment of the psoriasis-like model was defined by the appearance of epidermal hyperkeratosis and acanthosis and was further confirmed by histopathological examination of skin tissues.

### 2.2 Preparation of SYD and mice treatment

SYD is composed of Gypsum Fibrosum (Shigao), Arctii Fructus (Niubangzi), Rehmanniae Radix (Shengdihuang), Smilacis Glabrae Rhizoma (Baqia), Ophiopogonis Radix (Maidong), Moutan Cortex (Mudanpi), Paeoniae Radix Rubra (Chishao), Arnebiae Radix (Zicao), Platycodonis Radix (Jiegeng), Arctii Fructus (Niubangzi), Scrophulariae Radix (Xuanshen), Forsythiae Fructus (Lianqiao), Glycyrrhizae Radix et Rhizoma (Gancao), Lonicerae Japonicae Flos (Jinyinhua), Prunellae Spica (Xiakucao), and Scutellariae Radix (Huangqin). This formula has been granted a national invention patent in China (Patent No. ZL 2021 1 0056621.1). The sixteen herbs were mixed according to predefined proportions and extracted twice with boiling water (1:10, w/v) for 1 h per extraction cycle. The combined extracts were concentrated to obtain the final decoction. 24 h after the initial appearance of psoriasis-like phenotypes, the intervention protocol was initiated. Mice in the treatment group received SYD by oral gavage at a dose of 50 mL/kg/day. To maintain phenotypic stability during the intervention period, topical application of imiquimod cream was continued throughout the gavage treatment. Mice in the model group received an equivalent volume of distilled water by oral gavage and continued to receive daily imiquimod application. Visible regression of dorsal skin lesions was defined as the experimental endpoint, at which point the animals were euthanized and tissue samples were collected for subsequent analyses.

### 2.3 Phenotypic information collection and specimen collection of mice

Photographs of mice were taken daily using the same digital camera under identical lighting conditions and at a fixed distance. The severity of psoriasis-like lesions was quantitatively assessed using the Psoriasis Area and Severity Index (PASI) scoring system. Prior to each daily application of imiquimod cream, ear thickness was measured using a digital micrometer with a resolution of 0.01 mm. Body weight was recorded every 48 h. On day 7 after SYD gavage, when evidence of experimental endpoint was observed, mice were euthanized and samples were collected, including lesional dorsal skin, spleen, intestinal contents, and whole blood. Portions of splenic tissue and lesional skin were fixed in 4% paraformaldehyde (PFA; Sigma-Aldrich, P6148) for 24 h and embedded in paraffin (Leica, 39601006) for histopathological analysis. The remaining portions were snap-frozen in liquid nitrogen and stored at −80 °C for subsequent transcriptomic sequencing, intestinal contents were used for 16S rDNA sequencing and short chain fatty acid analysis.

### 2.4 Transcriptome sequencing and data analysis

Total RNA was extracted from snap-frozen mouse skin and splenic tissues using RNAiso Reagent (TaKaRa, 9108) according to the manufacturer's instructions. RNA integrity was assessed using a bioanalyzer, and samples with a RNA integrity number (RIN) > 7.0 and a 18S/28S ratio > 1.0 were considered qualified for downstream analysis. High-quality RNA was subsequently used for transcriptome library construction using the MGIEasy Fast RNA Reagent (MGI, 940-002921-00), mRNA was enriched using oligo (dT) magnetic beads, followed by fragmentation with fragmentation buffer at a controlled temperature. First-strand cDNA synthesis was performed according to the kit protocol, followed by second-strand cDNA synthesis. The resulting double-stranded cDNA underwent end repair and 3′-adenylation, after which sequencing adapters were ligated. Adapter-ligated products were amplified by PCR, and libraries that passed quality control were denatured into single-stranded DNA, circularized, and treated enzymatically to remove remaining linear DNA molecules. The single-stranded circular DNA was subjected to rolling circle amplification to generate DNA nanoballs (DNBs), which were loaded onto sequencing nanochips and sequenced on the DNBseq T7 platform using paired-end 150 bp reads.

Raw sequencing data were filtered using fastp (version: 0.23) (Chen et al., 2018) and subsequently aligned to the mouse reference genome (GRCm39: http://asia.ensembl.org/Mus_musculus/Info/Index) for gene quantification and downstream analyses. Differential gene expression analysis was performed using the DESeq2 package (version: 1.46.0), with an adjusted *p* value < 0.05 and fold change > 2 considered statistically significant. Gene Ontology (GO) and Kyoto Encyclopedia of Genes and Genomes (KEGG) enrichment analyses were conducted using the clusterProfiler package (version: 4.14.6), and gene set enrichment analysis (GSEA) was performed and visualized using the GseaVis package (version: 0.0.5).

CIBERSORT (https://cibersort.stanford.edu) was used for immune infiltration analysis, based on a reference dataset of gene expression characteristics of 22 known immune cell subtypes, calculate the types and distribution of various immune cells, and use ggplot2 R package to complete data statistics and result visualization.

### 2.5 Preparation of metagenomic 16S rDNA library

Microbial genomic DNA was extracted from an appropriate amount of snap-frozen intestinal content using the HiPure Stool DNA Kit B (Magen, D3141-01B) in accordance with the manufacturer's instructions. The integrity of genomic DNA was evaluated by 0.8% agarose gel electrophoresis, and DNA concentration was measured using Tecan F200 based on the PicoGreen fluorescence assay. The V3-V4 hypervariable region of bacterial 16S rDNA was amplified using region-specific primers: 314F (5′-CCTAYGGGRBGCASCAG-3′) and 806R (5′-GGACTACNNGGGTATCTAAT-3′). Sequencing libraries were constructed using the NEBNext Ultra II DNA Library Prep Kit for Illumina (NEB, E7645L). Paired-end sequencing (PE250) was performed on an Illumina NovaSeq 6000 platform using the NovaSeq 6000 SP Reagent Kit v1.5.

### 2.6 Analysis of metagenomic 16S rDNA rawdata

Paired-end reads were merged using FLASH (version 1.2.11), and subsequent quality control was performed with QIIME2 (version 2020.2) (Bolyen et al., 2019). The quality filtering criteria were as follows: sequences with an average quality score below 30 were removed; sequences shorter than 200 bp were discarded; and sequences containing any ambiguous bases (*N* > 0) were excluded. Sequence denoising and chimera removal were conducted using the Deblur algorithm, resulting in the generation of amplicon sequence variant (ASV) feature tables and representative sequences. The Naïve Bayes algorithm was used to construct the classifier of species classification dataset based on Silva database (version 138.2: http://www.arb-silva.de), which was subsequently used to annotate ASV representative sequences. To normalize sequencing depth across samples, rarefaction was conducted based on the minimum sequencing depth observed among all samples.

Alpha diversity analyses were performed using the vegan R package (version: 2.7-1). UniFrac distances were calculated using the GUniFrac package (version: 1.9), while Bray–Curtis and Jaccard distances were computed using the vegdist function in the vegan package. Principal coordinates analysis (PCoA) was conducted using the ape package (version: 5.8-1), and principal component analysis (PCA) as well as non-metric multidimensional scaling (NMDS) were performed using the vegan package. Hierarchical clustering analysis was carried out using the hclust function in the stats package. Analysis of similarities (ANOSIM) and permutational multivariate analysis of variance (PERMANOVA) were performed using the anosim and adonis functions of the vegan package, respectively. Community functional prediction was conducted using PICRUSt2 (version: 2.6.3) (Douglas et al., 2020), and differential taxonomic and functional analyses were performed using the lefser (version: 1.61.2) (Segata et al., 2011).

### 2.7 Short-chain fatty acid quantification

An appropriate amount of each sample was transferred into a centrifuge tube, followed by the addition of 50μL of 30% phosphoric acid and 300μL of acetone. The mixture was homogenized for 3 min and centrifuged at 12,000 rpm for 10 min. The supernatant was collected, diluted twofold, and used for subsequent analysis. Compound separation was performed using an Agilent gas chromatography system (Agilent, 7820) equipped with a DB-FFAP capillary column (30 m × 0.25 mm × 0.25μm). The injection volume was 1μL with a split ratio of 10:1, and high-purity helium was used as the carrier gas at a flow rate of 1.0 mL/min. The oven temperature was initially set at 70 °C and held for 5.0 min, followed by a temperature increase to 100 °C at a rate of 6 °C/min. Mass spectrometric detection was carried out using a quadrupole mass spectrometer (Agilent, 5977). The operating conditions were as follows: injector temperature, 260 °C; quadrupole temperature, 150 °C; acquisition mode, selected ion monitoring (SIM); and mass-to-charge (m/z) scan range of 30–550. Raw data were processed using Quant-My-Way (Software for MassHunter Workstation) for baseline correction, peak identification, and peak matching, and absolute quantification of compounds was performed using the external standard method.

### 2.8 Histopathology and immunohistochemistry

Fixed tissue specimens were sectioned at a thickness of 10 μm using a rotary microtome (Leica, RM2235). For hematoxylin and eosin (H&E) staining, sections were deparaffinized in xylene (Sigma-Aldrich, 214736) and rehydrated through a graded ethanol series. Sections were stained with Mayer's hematoxylin (Sigma-Aldrich, MHS16) for 5 min, followed by eosin staining (Sigma-Aldrich, HT110116) for 1 min, dehydrated, and mounted with DPX mounting medium (Sigma-Aldrich, 06522). Slides were imaged using a Nikon Eclipse E100 microscope.

For immunohistochemical analysis, antigen retrieval was performed by heating the sections in citrate buffer (pH 6.0; Abcam, ab93678) at 95 °C for 20 min. Sections were then sequentially treated with 3% hydrogen peroxide (Sigma-Aldrich, H1009) for 10 min to block endogenous peroxidase activity, followed by blocking with 5% bovine serum albumin (Sigma-Aldrich, A2153) containing 0.1% Triton X-100 for 30 min. Sections were incubated overnight at 4 °C with the following primary antibodies: anti-IL-17A (1:200; Abcam, ab79056; RRID), anti-tissue factor (Factor III) (1:150; Cell Signaling Technology, 98377; RRID), and anti-Ki67 (1:300; Abcam, ab16667; RRID). After washing, sections were incubated with an HRP-conjugated goat anti-rabbit IgG secondary antibody (1:500; Abcam, ab205718) for 1 h at room temperature. Immunoreactivity was visualized using a DAB substrate kit (Vector Laboratories, SK-4100), followed by counterstaining with Mayer's hematoxylin for 30 s. Image analysis was performed using ImageJ software (v1.53) with a threshold-based automated quantification approach.

### 2.9 Real-time quantitative PCR

Frozen tissue was transferred into a grinding tube containing 1mL of pre-chilled RNA extraction reagent (Wuhan Saiweier, G3013) and three 3mm grinding beads. Samples were thoroughly homogenized on ice, and total RNA was extracted according to the manufacturer's instructions. RNA concentration and purity were assessed using a NanoDrop 2,000 spectrophotometer. RNA concentration was diluted to a final concentration of 200 ng/μL. Reverse transcription was performed using a commercial reverse transcription kit (Wuhan Servicebio, G3337) in a 20μL reaction system for quantitative analysis. The primer sequences used are listed as follows: Mouse-Il4-F: CAGCTAGTTGTCATCCTGCTCTTC; Mouse-Il4-R: TCCCTTCTCCTGTGACCTCGTT

Mouse-Il17a-F: TCCACCGCAATGAAGACCCT; Mouse-Il17a-R: CATGTGGTGGTCCAGCTTTCC

Mouse-Gapdh-F: CCTCGTCCCGTAGACAAAATG; Mouse-Gapdh-R: TGAGGTCAATGAAGGGGTCGT. Relative gene expression levels were calculated using the comparative Ct (ΔΔCt) method, with normalization to GAPDH or β-actin expression.

### 2.10 ELISA

Whole blood collected in EDTA anticoagulant tubes was centrifuged at 3,000 rpm for 15 min at 2–8 °C. The supernatant was carefully transferred into a new 1.5 mL centrifuge tube and stored for future use. The Mouse IL-17A ELISA Kit (MultiSciences Biotech, EK217/2-48) was used for the assay following the manufacturer's instructions. A standard curve was first constructed using the concentration and OD values of the standard samples. Then, the OD values of the plasma samples were measured, and the concentration of IL-17A was calculated using the standard curve equation.

### 2.11 Statistical analysis

One-way analysis of variance (ANOVA) was used for multiple group comparisons, followed by Tukey's *post-hoc* test. For pairwise comparisons, the differences between two groups were analyzed using the one-sided Wilcoxon rank-sum test. Microbiome data were analyzed and visualized using QIIME2. Transcriptomic data analysis, as well as statistical analysis and data visualization of phenotypic data, were performed using R software (version 4.4.2). Correlation analysis was conducted using the Spearman method. *p* value < 0.05 was considered statistically significant.

## 3 Results

### 3.1 Animal modeling and experimental design route

**Figure 1:**
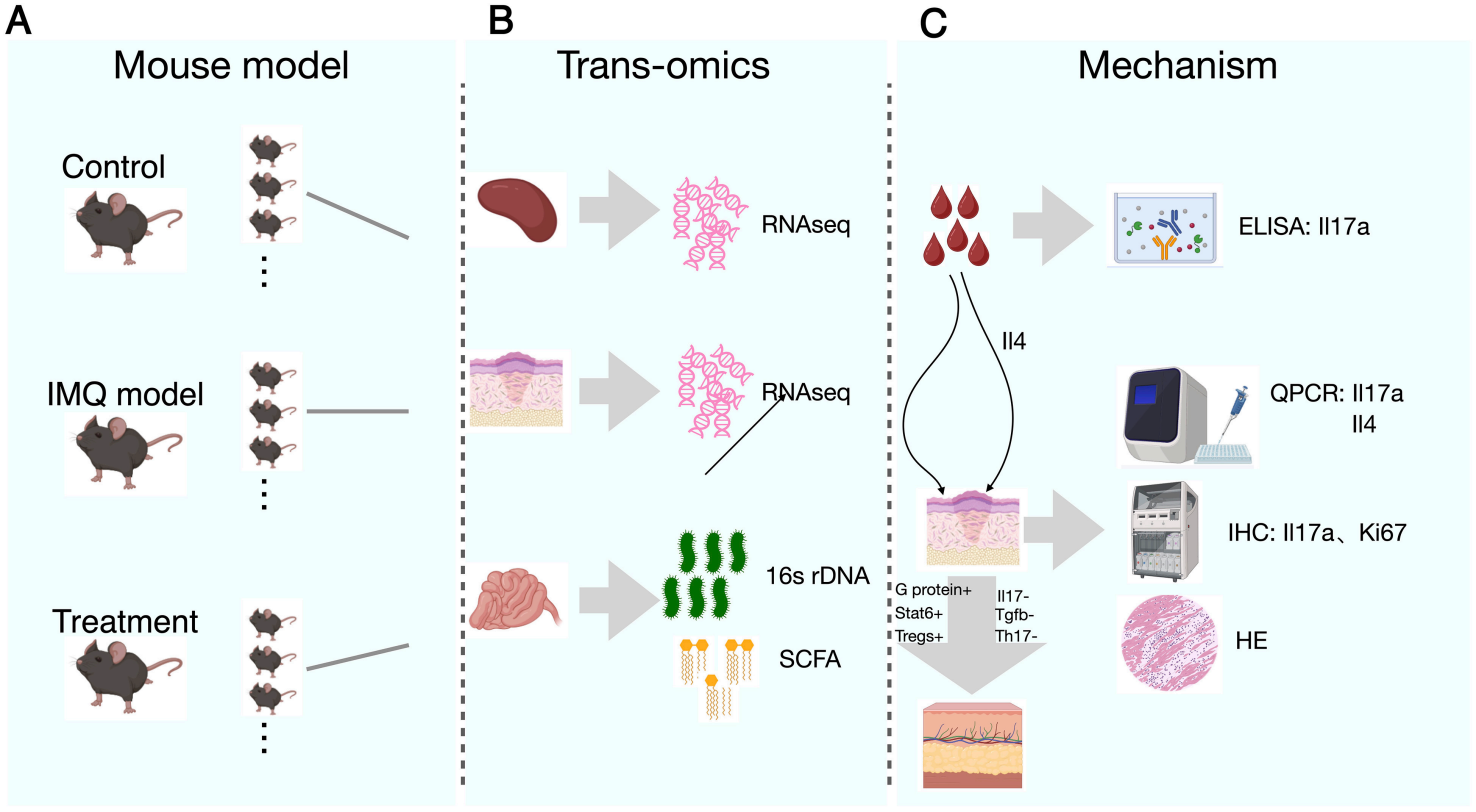
A schematic of the experiment. **(A)** The mouse model; **(B)** The multi omics data detection experiment; **(C)** The verification of key molecules.

Female BALB/c mice (6–8 weeks old) were divided into the healthy control group, psoriasis-like model group, and treatment group, as shown in Figure 1A. IMQ was administered until the mice developed skin lesions, as illustrated in Supplementary Figure 1, indicating successful establishment of the psoriasis-like model. Mice in the treatment group were gavaged with the SYD, and the skin lesions in the treated mice recovered to the state shown in Supplementary Figure 1, indicating the end of the treatment. Subsequent histopathological analysis of the skin tissues further confirmed the success of both the model and the SYD intervention. After the experiment, spleens, skin lesions, blood, and intestinal contents were collected from the mice. Spleen and skin samples were subjected to transcriptomic sequencing, while intestinal contents were analyzed by metagenomic 16S rDNA sequencing and short-chain fatty acid analysis. The graphical abstract of the experimental design was shown in Figure 1.

### 3.2 SYD alleviates psoriasis like phenotype and changes intestinal flora structure in mice

**Figure 2:**
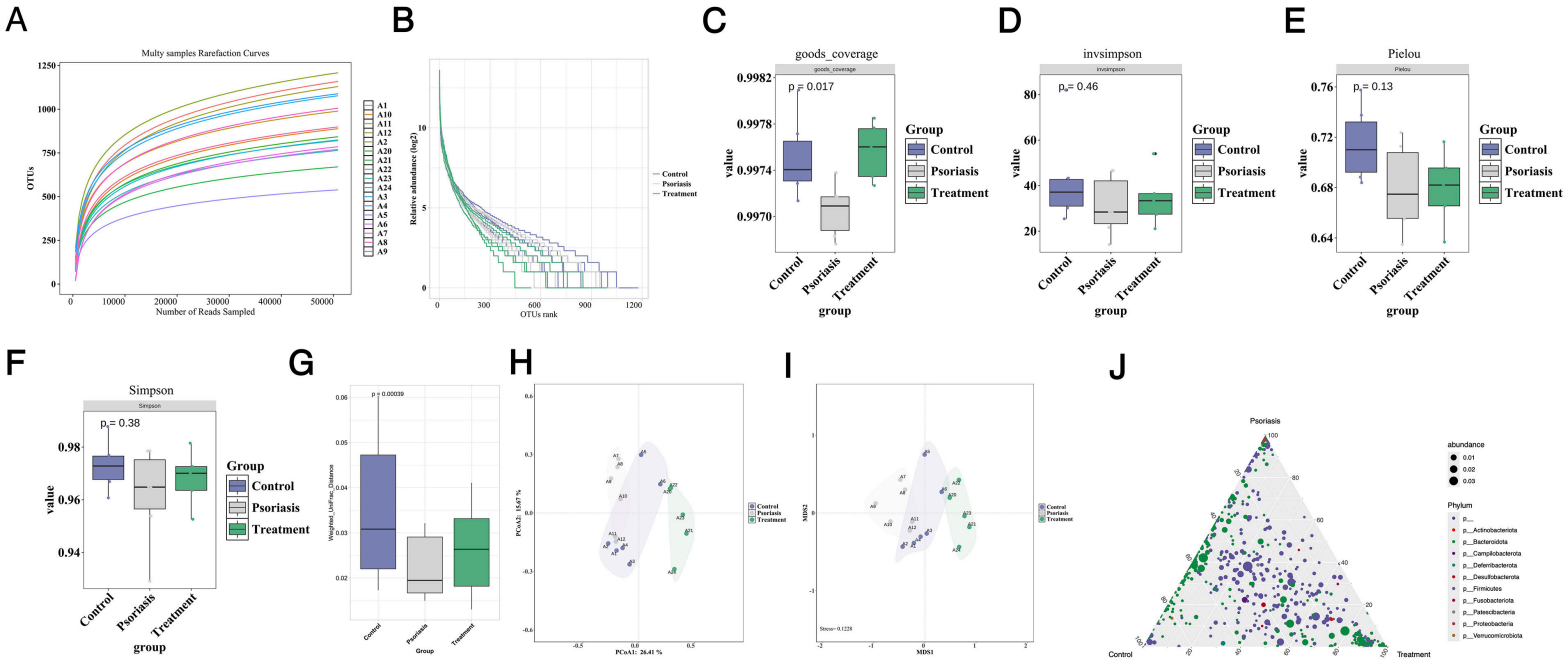
Changes in gut microbiota after Sanyin decoction treatment. **(A)** The rarefaction curves of samples; **(B)** The rank-abundance in different groups; **(C)-(F)** The alpha diversity in control group, psoriasis group and Treatment group, the alpha diversity indices were goods_coverage, invsimpson, Pielou, Simpson; **(G)** The Weighted_Unifrac_Distance between control group, psoriasis group and Treatment group; **(H)** The PCoA of samples in three groups; **(I)** The NMDS of samples in three groups; **(J)** The ternary phase diagram of three groups.

After the psoriasis-like mice were treated with SYD via gavage, significant improvement in skin lesions was observed, as shown in Supplementary Figure 1B. Concurrently, we performed 16S metagenomic sequencing on the intestinal contents of mice from the healthy control group, psoriasis model group, and treatment group. Figure 2A presents the species rarefaction curve, which shows that the number of operational taxonomic units (OTUs) increases slowly when the sequencing read count exceeds 5,000, indicating that the sequencing depth was sufficient for species identification. Figure 2B shows the rank-abundance curve, where a decrease in species evenness was observed after SYD treatment, the healthy control group had the highest species evenness, with the purple curve representing the control group.

We used the Vega software to calculate the alpha diversity indices (goods_coverage, insvsimpson, pielou, simpson) for the three groups, with results shown in Figures 2C–F. After SYD treatment, we found an increase in alpha diversity in the gut microbiota of the mice. Additionally, beta diversity analysis indicated significant differences in UniFrac distances between the groups (Figure 2G). PCoA and NMDS algorithms were applied to calculate the species distances between the three groups, with Figures 2H, I showing clear differences. We also analyzed the composition and distribution of dominant species among the three groups, as shown in Figure 2J. The microbial phyla p__Bacteroidota and p__Deferribacterota were predominantly distributed between the control and treatment groups, while p_Fusobacteriota was significantly enriched in the psoriasis model group.

### 3.3 SYD treatment enhances SCFA-producing bacteria in the gut of mice

**Figure 3:**
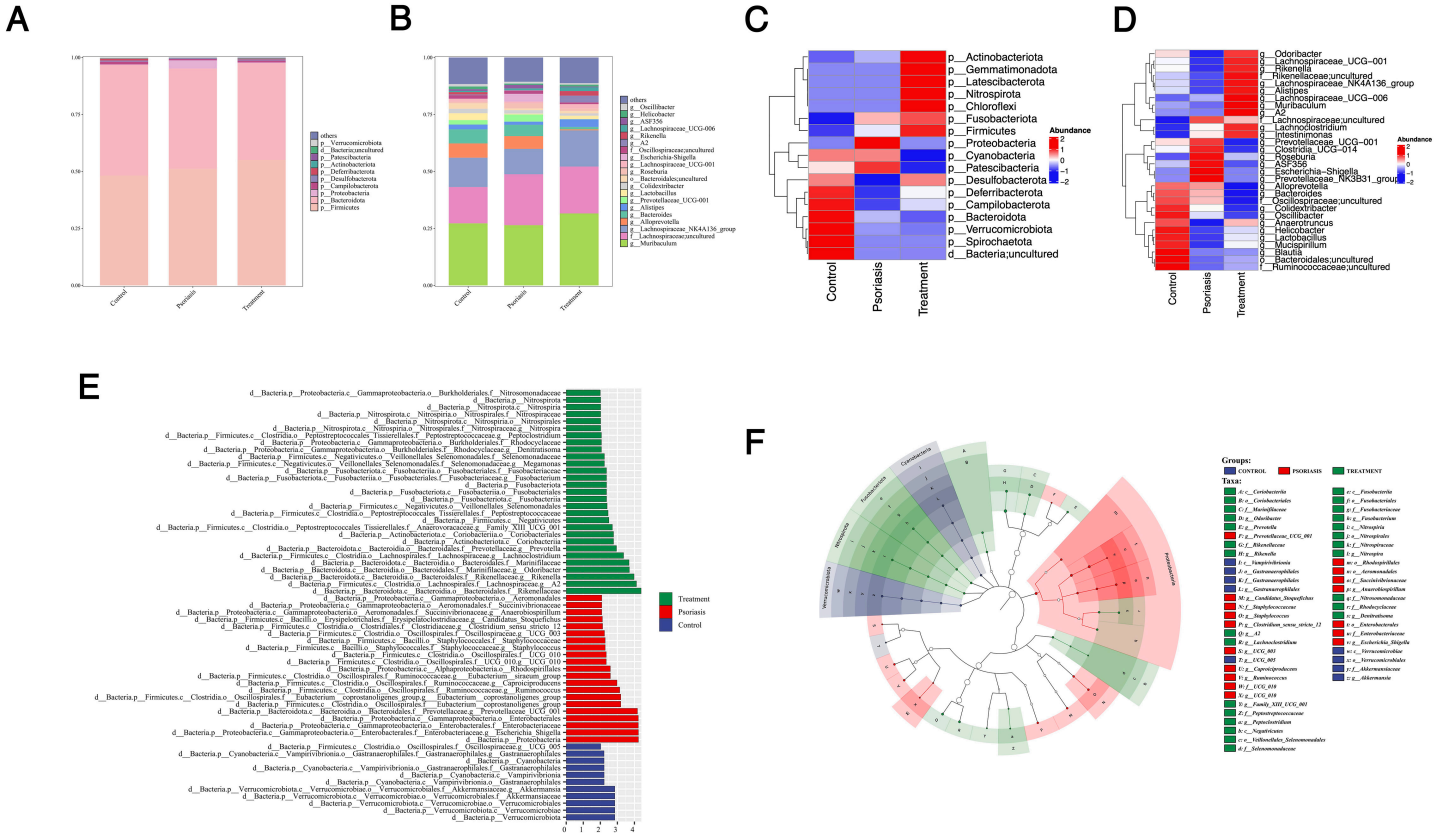
Structure and composition of gut microbiota in mice treated with Sanyin decoction. **(A)** The species composition at the phylum level; **(B)** The species composition at the genus level; **(C)** The heatmap of top 20 species at the phylum level; **(D)** The heatmap of top 30 species at the genus level; **(E)** and **(F)** The lefse analysis between three groups, **(E)** bar chart of LDA values, **(F)** branch diagram of species evolution.

Through microbiome composition analysis, we found that the abundance of *p__Proteobacterota, p__Patescibacteria*, and *p_Fusobacteriota* was increased in the psoriasis-like mouse model group. After treatment with traditional Chinese medicine, the abundance of *p__Firmicutes* and *p__Latescibacterota* was significantly elevated (Figures 3A, C). At the family and genus levels, the microbial composition is shown in Figures 3B, D. After treatment, the abundance of *g__Odoribacter, g__Lachnospiraceae_UCG-001, g__Rikenella, g__Lachnospiraceae_UCG-006*, and *g__Muribaculum* was significantly increased. LefSe analysis, using an LDA score > 2.5 and *p* < 0.05 to identify differentially abundant taxa, revealed that some microorganisms involved in the fermentation of short-chain fatty acids were significantly enriched in the treatment group, such as *g__Rikenella, g__Muribaculum*, and nitrifying bacteria like *p__Nitrospirota*. These anaerobic microorganisms, through fermentation, produce short-chain fatty acids and promote immune balance in the host (Figures 3E, F).

### 3.4 SYD treatment upregulates the abundance of SCFA in the gut microbiota

**Figure 4:**
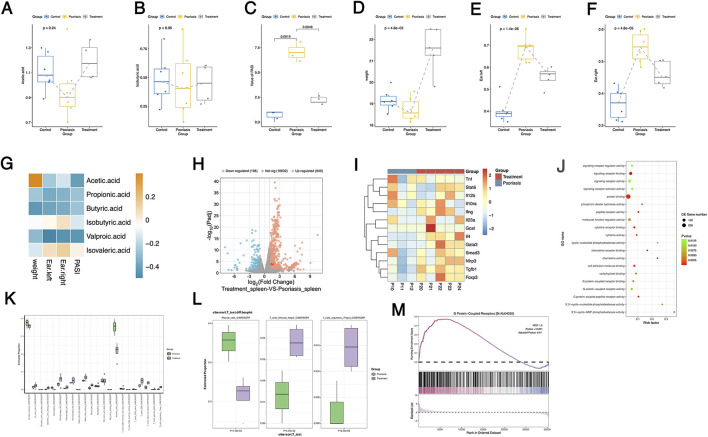
Changes in intestinal short chain fatty acids and spleen transcriptome in mice treated with SYD. **(A)** The boxplot of acetic acid abundance among three groups; **(B)** The boxplot of isobutyric acid abundance among three groups; **(C)** Differences in PISA scores among the three groups; **(D)** Differences in body weight among three groups of mice; **(E)** and **(F)** The difference in thickness between the left and right ears of mice among the three groups; **(G)** The correlation between short chain fatty acids and phenotype indicators in mice, yellow represents positive correlation, blue represents negative correlation, and darker colors indicate higher correlation coefficients; **(H)** The volcano plot of transcriptome between Psoriasis and SYD Treatment groups; **(I)** The heatmap of inflammation related genes among significant differentially expressed genes; **(J)** The dot plot of GO enrichment analysis of upregulated genes; **(K)** The GSEA analysis of the G protein-coupled receptor signaling pathway; **(L)** The analysis of CIBERSORT, which displayed the relative abundance of different cell subpopulations in three groups; **(M)** The box plot shows significant differences in the abundance of plasma, Treg, and Tfh cell subpopulations among the three groups.

Short-chain fatty acid (SCFA) levels in feces were measured, and the results showed that the abundance of acetate and butyrate decreased in the psoriasis group, but increased following treatment, although the differences between groups were not statistically significant (Figures 4A, B). Additionally, the PASI score was significantly higher in the psoriasis group, but decreased significantly after treatment, with a significant difference between groups (Figure 4C). Statistical analysis of body weight indicated a decrease in body weight in the model group, while the treatment group showed a trend of weight increase (Figure 4D). Figures 4E, F display changes in the thickness of the left and right ears of the mice. The ear thickness increased in the model group and decreased after treatment, with significant differences between groups as determined by ANOVA (Figures 4E, F). Moreover, we analyzed the correlation between the levels of acetate and butyrate and PASI scores and ear thickness, finding a negative correlation between the levels of acetate and butyrate and both PASI score and ear thickness (Figure 4G).

### 3.5 Remodeling of splenic immune status in mice following SYD treatment

After treating psoriasis-like mice with SYD, transcriptomic sequencing of spleen tissues revealed that 940 genes were significantly upregulated, while 198 genes were significantly downregulated (Figure 4H, Supplementary Table 1). Among these differentially expressed genes, the expression of inflammation-related genes among the differentially expressed genes is shown in Figure 4I, we found that Foxp3, Il4, Gata3, and Stat6 were highly expressed after treatment, while Tnf expression was decreased. The GO terms for upregulated genes were primarily enriched in signaling pathways such as G protein-coupled receptor binding/activity, 3′, 5′-cyclic GMP phosphodiesterase activity, and signaling receptor binding (Figure 4J). GSEA analysis revealed significant upregulation of the G protein-coupled receptor signaling pathway, which is consistent with the GO enrichment results (Figure 4M).

To investigate changes in the immune microenvironment of the spleen after treatment with the herbal formula, we used the CIBERSORT software with the deconvolution algorithm to calculate the relative abundance of different immune cell subpopulations. The relative abundance of all cell types is shown in Figure 4K. After treatment, the abundance of plasma cells decreased, while the abundance of follicular helper T cells and regulatory T cells was significantly increased in the treatment group (Figure 4L).

### 3.6 SYD promotes skin repair in psoriasis-like mice

**Figure 5:**
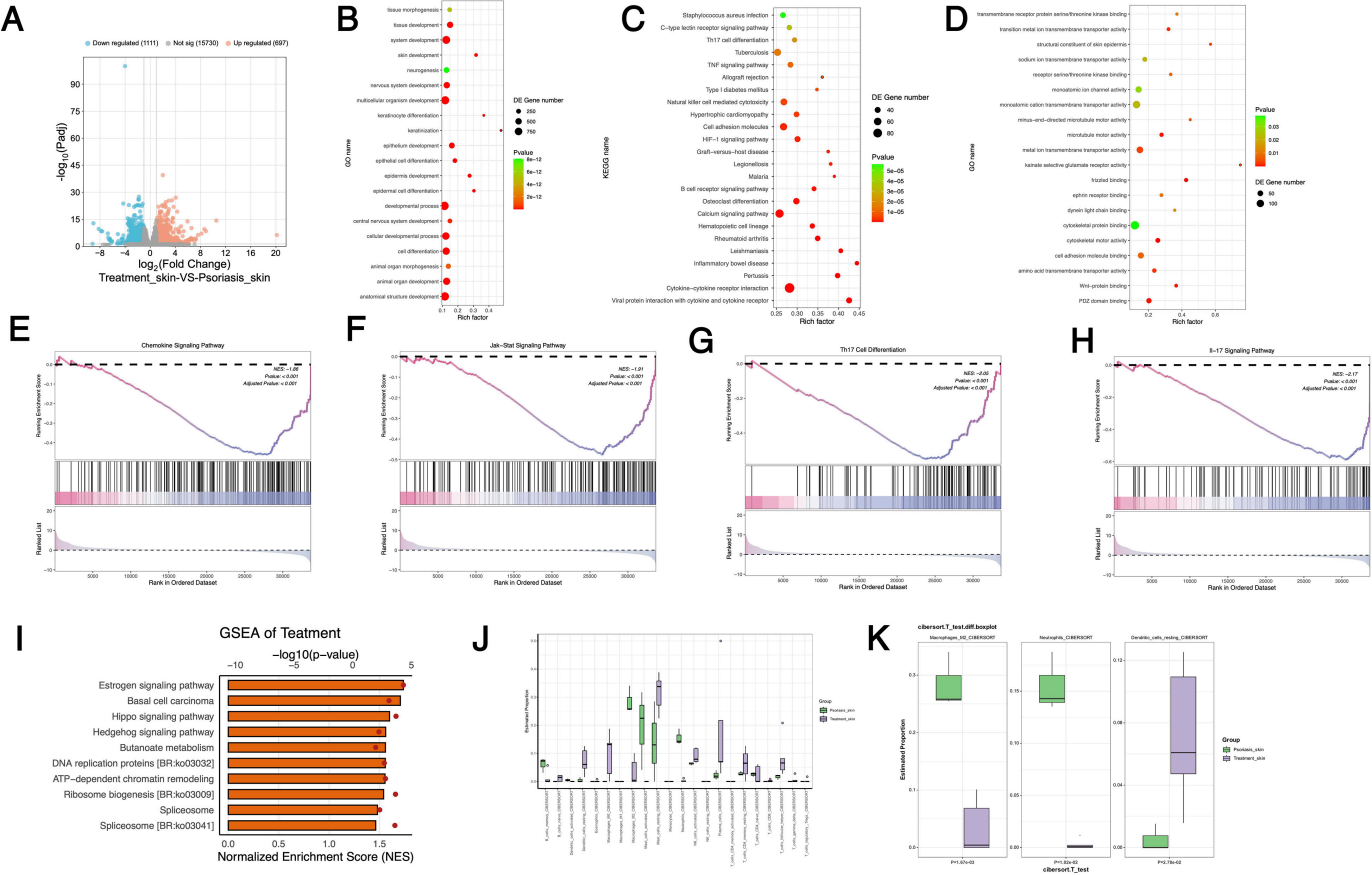
Changes in transcriptome of mouse skin after SYD treatment. **(A)** The volcano of differentially expressed genes; **(B)** The GO bioprocess (BP) analysis of significant upregulated genes; **(C)** The KEGG analysis of significant upregulated genes; **(D)** The GO molecular function (MF) analysis of significant upregulated genes; **(E)-(H)** The GSEA enrichment of significant regulated genes, NES < 0, represents downregulated signaling pathway, adjusted *p* value < 0.05, represents statistically significant; **(I)** The GSEA enrichment of upregulated signaling pathway; **(J)** The result of skin immune infiltration between Psoriasis group and Treatment group using CIBERSORT; **(K)** Boxplot of significant differences in immune cell subpopulations (Macrophage_M2, Neutrophils, Dendritic resting_cells).

After treating psoriasis-like mice with SYD, transcriptomic sequencing was performed on the skin lesion tissues. The results are shown in the volcano plot in Figure 5A, where 697 genes were significantly upregulated, and 1,111 genes were significantly downregulated (Supplementary table 2). Gene ontology (GO) enrichment analysis of upregulated genes revealed that the top 20 enriched terms (Figure 5B) included skin development, cell differentiation, and epithelial cell differentiation, all of which are associated with skin repair and cell differentiation. In the KEGG enrichment analysis of the top 20 downregulated genes, inflammation-related signaling pathways such as Th17 cell differentiation and TNF signaling pathway were significantly enriched (Figure 5C). Additionally, GO enrichment of upregulated genes revealed that terms directly related to skin repair were also significantly enriched, such as cell adhesion molecule binding, frizzled binding/Wnt-protein binding, metal ion transmembrane transporter activity, and sodium ion transmembrane transporter activity (Figure 5D).

Gene set enrichment analysis (GSEA) showed that the Chemokine signaling pathway, Jak-Stat signaling pathway, and Th17 cell differentiation signaling pathways were significantly downregulated (Figures 5E–H). Notably, the Hippo signaling pathway, Hedgehog signaling pathway, Estrogen signaling pathway, and Basal cell carcinoma signaling pathways were significantly upregulated (Figure 5I). Analysis of the relative abundance of different cell populations in the skin tissue revealed that the relative abundance of Macrophage M2 and Neutrophils was significantly reduced after treatment, while the abundance of Dendritic_cells was significantly increased (Figures 5J, K).

### 3.7 SYD treatment modulates splenic immune gene expression, upregulates IL-4, and promotes Th2-related immune signaling

Transcriptomic analysis of splenic tissue revealed significant transcriptional changes in psoriasis-like mice following SYD treatment, with a notable enrichment of immune-related signaling pathways (Figures 5B–D). Among the differentially expressed cytokine genes, Il4 expression was significantly upregulated in the treatment group compared to the psoriasis model group (Figure 4I).

**Figure 6:**
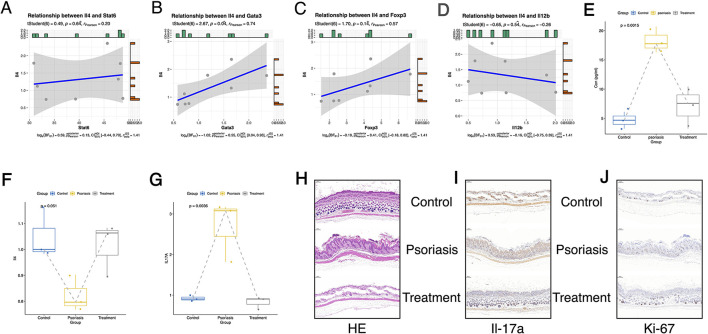
Psoriasis-like skin in mice were improved after SYD treatment. **(A)** The correlation between Il4 and Stat6; **(B)** The correlation between Il4 and Gata3; **(C)** The correlation between Il4 and Foxp3; **(D)** The correlation between Il4 and Il2b; **(E)** Detection of Il17a levels in mouse serum using Elisa; **(F)** Il4 gene expression in control mouse skin, psoriasis-like mouse skin, and SYD treated mouse skin using QPCR; **(G)** Il17a gene expression in control mouse skin, psoriasis-like mouse skin, and SYD treated mouse skin using QPCR; **(H)** HE results of Control group, Psoriasis group and Treatment group, Scale bars = 200μm; **(I)** Expression levels of Il17a in Control group, Psoriasis group and Treatment group were evaluated by immunohistochemical analysis, Scale bars = 200μm; **(J)** Expression levels of Ki-67 in Control group, Psoriasis group and Treatment group were evaluated by immunohistochemical analysis, Scale bars = 200μm.

Further correlation analysis indicated that the expression of Il4 was positively correlated with key transcription factors involved in Th2-related immune signaling, including Stat6 and Gata3, as well as the regulatory *T* cell marker Foxp3 (Figures 6A–C). In contrast, Il4 expression showed a negative correlation with Il12b (Figure 6D), a cytokine associated with pro-inflammatory Th1/Th17 responses. These coordinated transcriptional associations suggest that Il4 may play a central role in the splenic immune gene expression network regulated by SYD.

In addition, we also validated the changes in key cytokines in mouse skin and blood. Quantitative PCR (qPCR) confirmed the differential expression of Il4 and Il17a, which was consistent with the transcriptomic sequencing data (Figures 6F, G). The relative abundance of Il17a in plasma was reduced after treatment with SYD (Figure 6E). Histopathology showed that SYD treatment reduced skin scars and hyperplasia in mice (Figure 6H). Immunohistochemical analysis of skin sections further demonstrated a marked decrease in Il17a and Ki-67 protein levels after administration of the SYD (Figures 6I, J).

### 3.8 Proposed mechanism by which SYD alleviates psoriasis through modulation of the gut-spleen-skin axis

**Figure 7:**
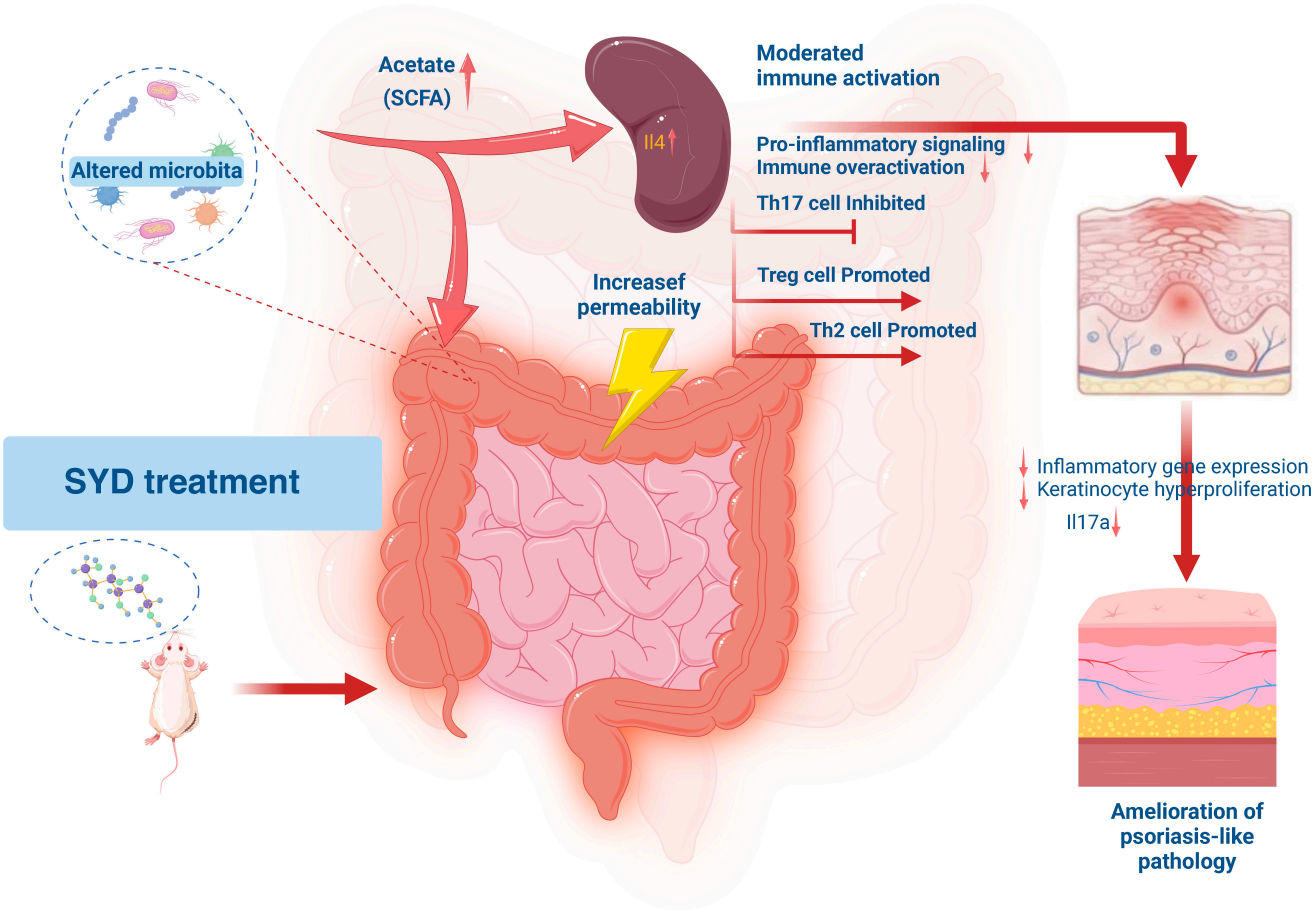
The proposed mechanism: SYD alleviates psoriasis-like inflammation via the gut microbiota–acetate–spleen axis. We referenced the schematic template from the website https://aidraw.cnsknowall.com/ and created this diagram based on the results of our study.

Based on the above research, we proposed a mechanism hypothesis for SYD to alleviate psoriasis, SYD treatment reverses gut microbiota dysbiosis in IMQ-induced mice, notably increasing the abundance of acetate-producing bacteria and fecal acetate levels (Figure 7). Elevated circulating acetate is proposed to act on the spleen, suppressing excessive immune activation and reprogramming the splenic transcriptome toward an anti-inflammatory profile. This systemic regulation subsequently reduces cutaneous inflammation and keratinocyte hyperproliferation. Note: This diagram summarizes potential pathways supported by multi-omics associations observed in this study; direct causal relationships remain to be fully elucidated.

## 4 Discussion

Increasing evidence suggested that changes in gut microbiota composition can influence systemic immune responses through the production of microbiota-derived metabolites (Yang and Cong, 2021; Rooks and Garrett, 2016). In this study, treatment with SYD was significantly associated with the enrichment of bacterial genera that produce short chain fatty acids, including members of *Lachnospiraceae, Rikenella*, and *Muribaculum*, accompanied by an increase in fecal short-chain fatty acid levels, particularly acetate. These findings are consistent with previous reports describing a reduced capacity for SCFA production in inflammatory and autoimmune diseases, including psoriasis, and support the concept that the restoration of microbial metabolic output may be related to immune homeostasis (Kim, 2023; Duscha et al., 2020).

Among the short-chain fatty acids (SCFAs), acetate is the most abundant and systemically available metabolite, readily entering circulation and exerting immune regulatory effects beyond the gut (Fukuda et al., 2011; Kau et al., 2011). Previous studies have shown that acetate can influence immune cell activation, cytokine production, and regulatory T cell function through various mechanisms, including the involvement of G protein-coupled receptors and the modulation of intracellular signaling pathways (Tan et al., 2017; Correa-Oliveira et al., 2016; Zhao et al., 2025). Consistent with these findings, our transcriptomic analysis revealed the activation of G protein-coupled receptor-related signaling pathways in the spleen following SYD treatment, suggesting a potential link between increased acetate utilization and splenic immune signaling regulation.

As a central immune organ, the spleen integrates circulating metabolism and immune signals and coordinates the downstream immune response. Emerging evidence suggests that gut microbiota-derived metabolites can shape the composition and functional state of splenic immune cells, thereby influencing systemic immune balance (Yang and Cong, 2021; Wang et al., 2023; Rooks and Garrett, 2016). The simultaneous increase in acetate levels and IL-4–associated signaling observed in the spleen in our study supports a model in which gut microbial metabolic remodeling may be linked to splenic immune reprogramming (Sun et al., 2020; Stienstra et al., 2017; Dong et al., 2025). Notably, this association does not imply a direct causal relationship, but rather suggests that acetate may help create an immune environment conducive to regulatory immune responses. SYD treatment was associated with increased IL-4 expression in the spleen and elevated plasma IL-4 levels. Although IL-4 is typically linked to Th2 immunity, Th2-associated cytokines have been reported to antagonize Th17 differentiation and/or IL-17–mediated responses, suggesting a potential counter-regulatory effect that may contribute to the suppression of IL-17 signaling observed in lesional skin. Nevertheless, our data are primarily associative, and further studies are required to determine whether IL-4 is mechanistically required for the downstream attenuation of Th17 inflammation.

Importantly, the spleen-mediated immune regulation observed in this study was accompanied by the attenuation of inflammatory signaling pathways in the skin, including the suppression of the IL-17 pathway and the activation of keratinocyte differentiation-related programs. These findings align with the concept that systemic immune regulation, rather than localized skin intervention, plays a key role in controlling psoriasis-associated inflammation (Vetrani et al., 2022; Pan et al., 2025). Within this framework, the gut–spleen–skin axis provides a coherent explanation of how changes in the gut microbiota can translate into immune outcomes in distant tissues.

In summary, this study provides an integrated perspective on the immunomodulatory effects of SYD in a psoriasis-like mouse model by linking gut microbiota remodeling, functional metabolite output, and peripheral immune regulation. Our findings do not attribute the therapeutic effects to a single molecular target but rather emphasize the synergistic interaction of the gut microbiota–short-chain fatty acids–spleen–skin axis, with microbial-derived acetate and IL-4–associated immune signaling acting as interconnected components of systemic immune rebalancing. Although the current study is limited by its reliance on associative analysis and does not establish a direct causal relationship between gut-derived metabolites and immune modulation, the integration of microbiome analysis, metabolomics, and transcriptomics in immune-related tissues supports the biological plausibility of this multi-organ regulatory framework. This systems-level perspective not only advances the understanding of psoriasis as a disease involving distal immune crosstalk but also offers a generalizable strategy for studying the mechanisms of traditional herbal formulas in complex inflammatory diseases.

## 5 Conclusion

All in all, our study reveals that SYD can improve the phenotype of psoriasis like mice by coordinating the regulation of gut spleen cutaneous axis, which is characterized by restoring intestinal microbial balance, increasing microbial acetate, and systemic immune reprogramming. These changes are accompanied by spleen immune activation and inhibition of skin inflammatory reaction, which jointly promote the improvement of the disease. This comprehensive framework provides a biologically reasonable explanation of how multi-component herbal formulas exert therapeutic effects through microbiota immune skin crosstalk.

## Data Availability

The datasets of bulk RNAseq and 16s rDNA sequence for this study can be found in the NCBI SRA database (PRJNA1377919): https://www.ncbi.nlm.nih.gov/bioproject/PRJNA1377919.
