# Supplementary figures and images for: Sanyin decoction alleviates psoriasis by reshaping gut microbiota and modulating the gut–spleen–skin axis
Source: Front Microbiol. 2026 Apr 16;17:1799928. doi: 10.3389/fmicb.2026.1799928 (PMC13132127; doi:10.3389/fmicb.2026.1799928)

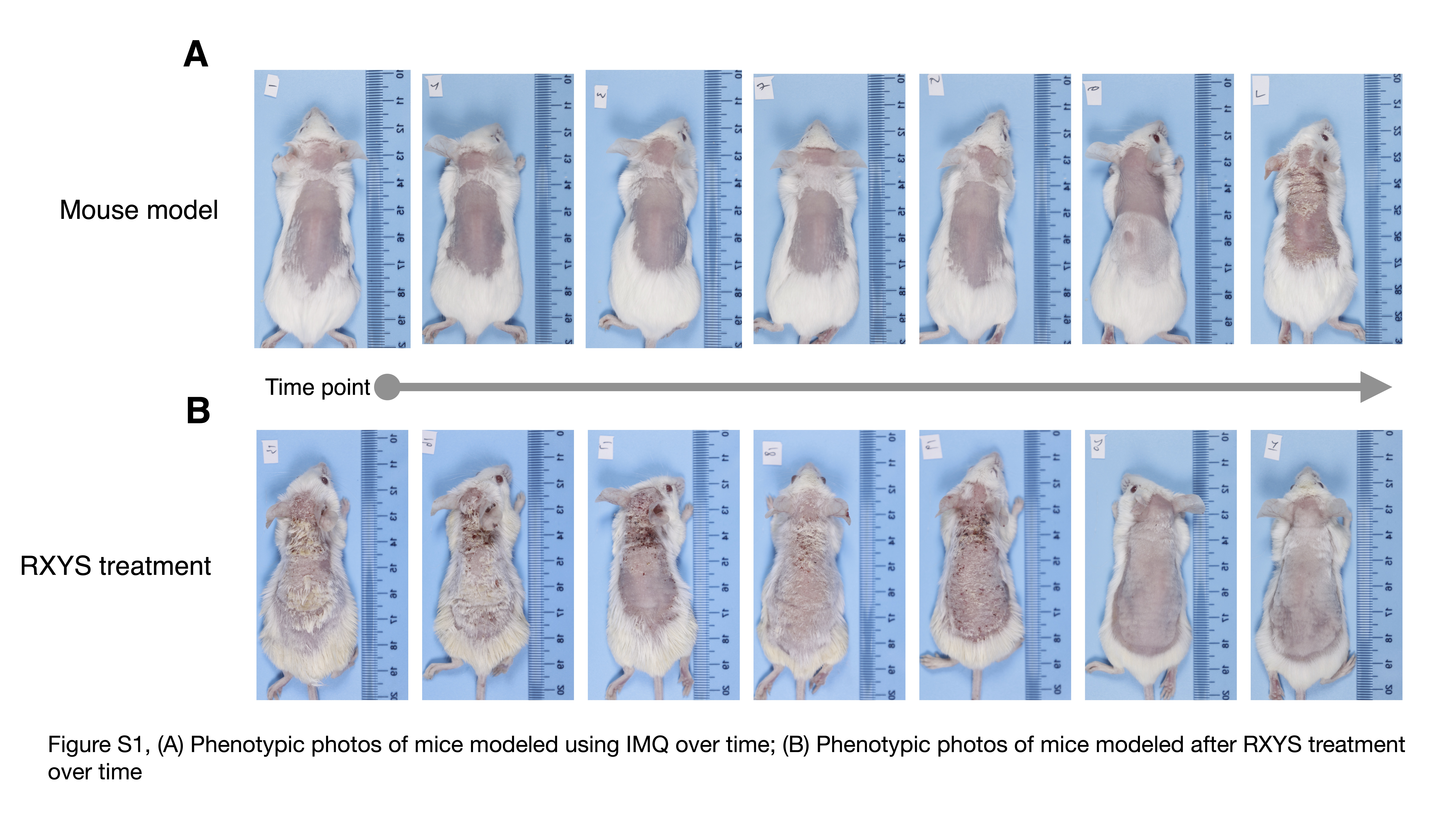

Supplement: Supplementary file 3 [file Image_1.jpeg]
